# TULP1 and TUB Are Required for Specific Localization of PRCD to Photoreceptor Outer Segments

**DOI:** 10.3390/ijms21228677

**Published:** 2020-11-17

**Authors:** Lital Remez, Ben Cohen, Mariela J. Nevet, Leah Rizel, Tamar Ben-Yosef

**Affiliations:** 1 Ruth & Bruce Rappaport Faculty of Medicine, Technion-Israel Institute of Technology, Haifa 31096, Israel; litalremez@gmail.com (L.R.); i916bc@gmail.com (B.C.); judithpeist@gmail.com (M.J.N.); rizel@technion.ac.il (L.R.); 2 Department of Dermatology, Rambam Health Care Campus, Haifa 3109601, Israel

**Keywords:** retina, photoreceptors, photoreceptor disc component, TULP1, TUB

## Abstract

Photoreceptor disc component (PRCD) is a small protein which is exclusively localized to photoreceptor outer segments, and is involved in the formation of photoreceptor outer segment discs. Mutations in PRCD are associated with retinal degeneration in humans, mice, and dogs. The purpose of this work was to identify PRCD-binding proteins in the retina. PRCD protein-protein interactions were identified when implementing the Ras recruitment system (RRS), a cytoplasmic-based yeast two-hybrid system, on a bovine retina cDNA library. An interaction between PRCD and tubby-like protein 1 (TULP1) was identified. Co-immunoprecipitation in transfected mammalian cells confirmed that PRCD interacts with TULP1, as well as with its homolog, TUB. These interactions were mediated by TULP1 and TUB highly conserved C-terminal tubby domain. PRCD localization was altered in the retinas of TULP1- and TUB-deficient mice. These results show that TULP1 and TUB, which are involved in the vesicular trafficking of several photoreceptor proteins from the inner segment to the outer segment, are also required for PRCD exclusive localization to photoreceptor outer segment discs.

## 1. Introduction

Photoreceptors are sensory cells which generate electrical signals in response to light stimuli. These cells are highly compartmentalized, with the nucleus and other cellular organelles located in the inner segment (IS), and the entire machinery for the visual signal transduction cascade (phototransduction) included in the outer segment (OS). Photoreceptor OS are highly modified primary sensory cilia characterized by thousands of stacked disc membranes. The proximal end of the OS is linked to the cell body (i.e., the IS) via a connecting cilium, which is structurally homologous to the transition zone of primary cilia. Unique OS proteins are formed in the IS and transported to the OS across the connecting cilium (reviewed in reference [1]). Outer segments are continually renewed throughout the lifetime of the vertebrate retina. New discs are formed at the OS base, while old discs are shed from the OS tip [2,3].

Photoreceptor disc component (PRCD) (MIM #610598) is a small protein (54 amino acids in dogs and humans and 53 amino acids in mice), which is exclusively localized to OS discs in both rods and cones [4,5]. Localization of PRCD to OS membranes requires N-terminal post-translational modifications, including S-acylation and palmitoylation [6,7]. PRCD is concentrated at the OS rim, where it is present in both the disc membrane and the photoreceptor plasma membrane [5]. Interestingly, PRCD is highly concentrated at the base of the OS, the site of disc neo-genesis [5]. Indeed, recently, it was shown that PRCD is essential for high-fidelity OS disc formation [5,8]. Mutations in *PRCD* cause progressive rod-cone degeneration (i.e., retinitis pigmentosa (RP)) in humans, dogs, and mice [5,8,9,10].

Here, we describe the use of the Ras recruitment system (RRS), a cytoplasmic-based yeast two-hybrid system [11], to identify PRCD-interacting proteins in the retina. We identified an interaction of PRCD with tubby-like protein 1 (TULP1) and with TUB. TULP1 and TUB are members of the tubby-like family of proteins, including TUB, TULP1-3, and the more distantly related TULP4. TULP proteins are defined by the highly conserved ‘tubby domain’ located in their C-terminal part, which binds selectively to specific membrane phosphoinositides. The N-terminus is diverse and directs distinct functions [12]. TULP1 and TUB are highly expressed in the retina, and mutations in both of them are associated with progressive retinal degeneration in humans and in mice [13,14,15,16,17]. Both TULP1 and TUB are involved in the vesicular trafficking of photoreceptor proteins from the IS to the OS [15,18,19,20]. In *Tulp1*-knockout mice, several OS proteins, including rhodopsin, guanylate cyclase (GC1), guanylate cyclase-activating proteins 1 and 2 (GCAP1 and GCAP2, respectively), and blue cone opsin, are mislocalized, and found throughout the photoreceptor, as opposed to being exclusively localized to the OS [18]. In addition, the transition of the arrestin signaling protein from the IS to the OS in response to light is disrupted in these mice [18]. Mislocalization of rhodopsin and of cone opsins was also observed in TUB-deficient mice [20]. We therefore characterized the interaction of TULP1 and TUB with PRCD, and its effect on PRCD’s specific localization to photoreceptor OS.

## 2. Results

### 2.1. PRCD Interacts with Both TULP1 and TUB

**Figure 1:**
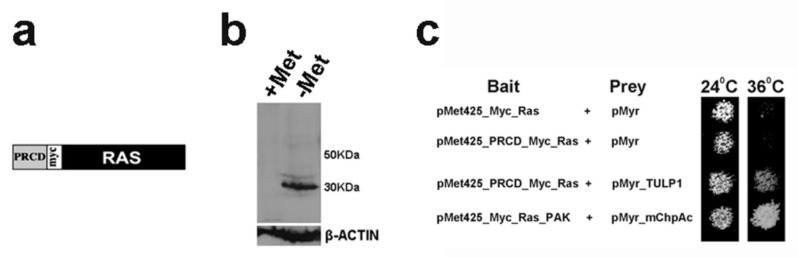
Identification of photoreceptor disc component (PRCD) interaction with TULP1 in yeast. (**a**) pMet425-PRCD-Myc-Ras bait construct used for the Ras recruitment system (RRS). The construct encodes for a chimeric protein, composed of mouse PRCD fused to a cytoplasmic Ras mutant, with a Myc tag at the C-terminus. (**b**) Western blot analysis of yeast transformed with the pMet425-PRCD-Myc-Ras bait construct, with an anti-Myc tag antibody, showing a 30 kDa band in yeast grown on media lacking methionine (−Met). No band appears in the lane containing extract from yeast grown on methionine rich media (+Met), indicating inducibility of the pMET425 promotor. (**c**) *cdc25-2* yeast were co-transformed with the indicated bait and prey combinations, and grown on GAL–LUM plates incubated in the permissive (24 °C) and the restrictive (36 °C) temperatures. Co-transformations of yeast with empty bait and prey vectors (1st row) or with the PRCD-bait vector and an empty prey vector (2nd row) served as negative controls. Pak and ChpAc are two proteins known to interact with each other [21], and thus served as a positive control (4th row). Yeast transformed with a combination of PRCD and TULP1 (3rd row) grew at both the permissive and the restrictive temperatures, therefore indicating an interaction between these proteins.

To identify potential binding partners of PRCD, we used the RRS [11]. We generated a bait construct (pMet425-PRCD-Myc-Ras), which encodes for a chimeric protein, composed of mouse PRCD with a C-terminal Myc tag fused to a cytoplasmic Ras mutant (Figure 1a). The chimeric protein was expressed in yeast in media lacking methionine (Figure 1b), and did not allow growth of *cdc25-2* yeast at 36 °C by itself (Figure 1c, 2nd row). This bait construct was used for an RRS screen of a bovine retinal cDNA expression library. In the RRS system, complementation of the *cdc25-2* strain is achieved through Ras membrane localization and activation due to interaction between two hybrid proteins [11].

A total of six clones, representing putative binding partners of PRCD, were identified. Sequencing analysis revealed that four of the clones were identical, all derived from bovine *TULP1* cDNA (NM_001206728.1) and encoding for the C-terminus of the protein (amino acids 220-546). The combination of these prey clones with a PRCD bait construct enabled growth of *cdc25-2* yeast on GAL-LUM media, at both permissive (24 °C) and restrictive (36 °C) temperatures (Figure 1c, 3rd row). In this media, both bait and prey proteins were expressed. The same plasmid combination did not allow growth of *cdc25-2* yeast at the restrictive temperature on GAL-LU or on GLU-LUM media, on which only the prey protein or the bait protein were expressed, respectively (data not shown). These results indicated that the identified TULP1 prey protein did not complement the *cdc25-2* mutation by itself, and that complementation was achieved only due to its interaction with PRCD.

**Figure 2:**
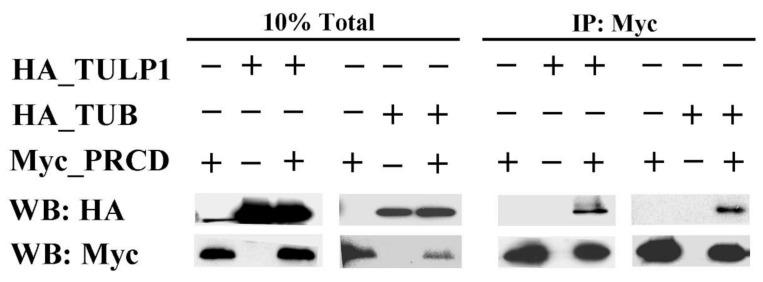
Verification of the interaction between PRCD, TULP1, and TUB by co-immunoprecipitation (co-IP). COS-7 cells were transiently co-transfected with different combinations of PRCD_Myc, TULP1_HA, and TUB_HA. Cell lysates were subjected to co-IP with an anti-Myc tag antibody, and eluted proteins as well as total cell lysates were separated by SDS-PAGE, followed by Western blotting (WB) with anti-HA and anti-Myc tag antibodies. The presence of TULP1 and TUB in the anti-Myc tag immunocomplexes supported their interaction with PRCD in mammalian cells.

The interaction of PRCD with TULP1 was further tested by co-immunoprecipitation (co-IP), using lysates from COS-7 cells expressing Myc-tagged PRCD and HA-tagged full-length mouse TULP1. The presence of TULP1 in the anti-Myc (PRCD) immunocomplex supported its interaction with PRCD in mammalian cells (Figure 2). We then used co-IP to test for possible interaction between PRCD and TUB, due to its close homology to TULP1. TUB was also shown to interact with PRCD (Figure 2).

### 2.2. PRCD’s Interaction with TULP1 and TUB Is Mediated by the Highly Conserved Tubby Domain

**Figure 3:**
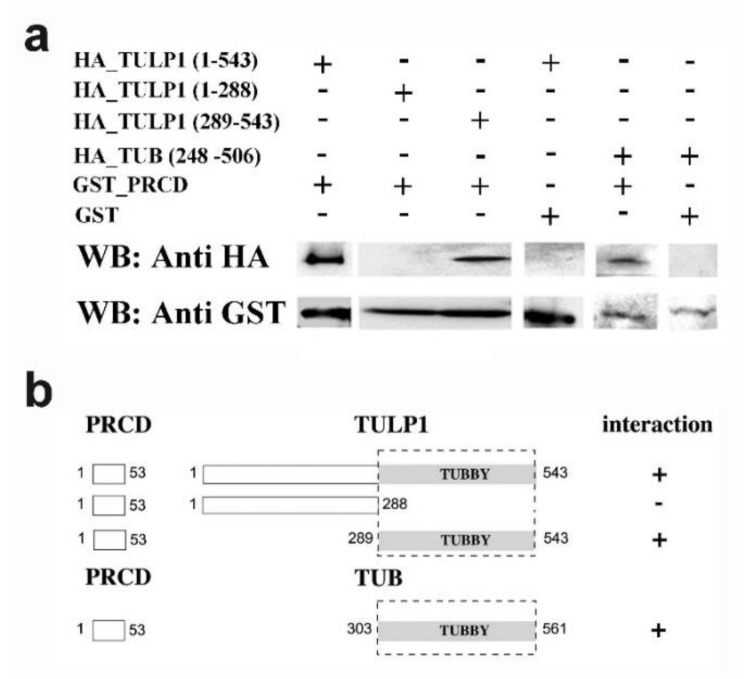
Mapping the interacting regions between PRCD, TULP1, and TUB. (**a**) Pull-down experiments were performed to test the interaction between PRCD_GST and truncated TULP1_HA and TUB_HA. Samples were analyzed by Western blot with anti-HA and anti-GST antibodies. Within TULP1, the interacting region was mapped to the C-terminal part of the protein (aa 289-543), the region which includes the tubby domain. The N-terminal part of the protein (aa 1-288) did not interact with PRCD. The tubby domain of TUB (aa 248-506) also interacted with PRCD. (**b**) Shown is a schematic representation of the different pull-down experiments performed to map the interacting regions. The scattered line represents the detected interacting parts.

To map the interacting regions in each protein, we performed pull-down experiments, using truncated TULP1_HA or TUB_HA and purified PRCD_GST beads. Within TULP1, the interacting region was mapped to the C-terminal part of the protein, the region which includes the highly conserved tubby domain (Figure 3a,b). The tubby domain of TUB also interacted with PRCD (Figure 3a,b).

**Figure 4:**
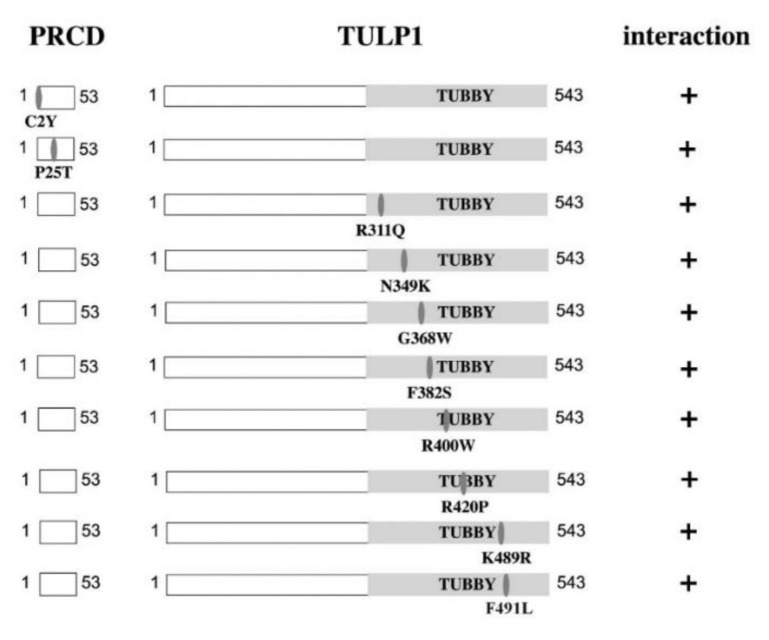
The effect of mutations in PRCD and TULP1 on their interaction. Shown are schematic representations of the different PRCD and TULP1 mutated constructs tested. Each line represents a pull-down experiment. An interaction between PRCD and TULP1 was observed for all tested mutations.

We next used pull-down experiments to test the effect of the following missense mutations (detected in human patients) on PRCD-TULP1 interaction: PRCD: p.C2Y and p.P25T [9,22]; TULP1: p.R311Q, p.N349K, p.G368W, p.F382S, p.R400W, p.R420P, p.K489R, and p.F491L (all located within the tubby domain) [16,23,24,25,26,27]. An interaction between PRCD and TULP1 was observed for all tested mutations (Figure 4). It should be noted, however, that some mutations might affect the interaction in a quantitative rather than a qualitative manner, and that this aspect was not well assessed by the pull-down assay.

### 2.3. PRCD Is Mislocalized in Photoreceptors of TULP1- and TUB-Deficient Mice

**Figure 5:**
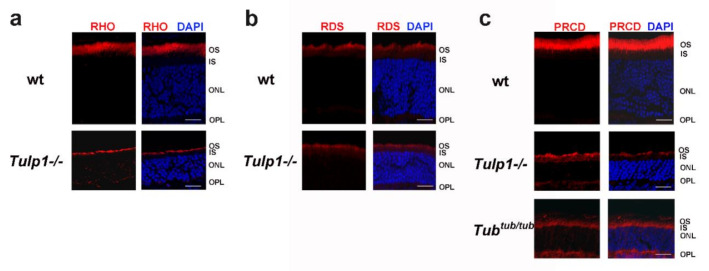
PRCD is mislocalized in photoreceptors of *Tulp1*^-/-^ and *Tub^tub/tub^* mice. (**a**) Immunostaining of retinal sections derived from wt and *Tulp1*^-/-^ mice, with an antibody against rhodopsin (RHO) (red). (**b**) Immunostaining of retinal sections derived from wt and *Tulp1*^-/-^ mice, with an antibody against peripherin/RDS (red). (**c**) Immunostaining of retinal sections derived from wt, *Tulp1*^-/-^, and *Tub^tub/tub^* mice, with an antibody against PRCD (red). DAPI served for nuclear staining (blue). scale bars: 20 μm. IS, inner segment; ONL, outer nuclear layer; OPL, outer plexiform layer; OS, outer segment.

Both TULP1 and TUB are involved in the vesicular trafficking of photoreceptor proteins from the IS to the OS. PRCD is a unique photoreceptor disc component. We therefore tested whether its interaction with TULP1 and/or TUB is required for its specific localization to the OS. For this purpose, we performed immunostaining of retinal sections obtained from TULP1- and TUB-deficient mice and wt controls at post-natal day 21. As a positive control, we used rhodopsin, which was previously shown to be mislocalized in photoreceptors of *Tulp1*-knockout mice (Figure 5a) [18]. As a negative control, we used peripherin/RDS, which was previously shown to be unaffected by the lack of TULP1, and is therefore properly localized to OS in both wt and *Tulp1*-knockout mice (Figure 5b) [18]. Similar to rhodopsin, in wt mice, PRCD was exclusively located to photoreceptor OS, while in *Tulp1*-knockout mice, it was found in the OS, IS, outer nuclear layer, and outer plexiform layer (Figure 5c). PRCD mislocalization was also found in photoreceptors of *Tub* mutant mice (Figure 5c).

## 3. Discussion

PRCD is a protein of crucial importance for normal retinal function. This fact was established over a decade ago, with the identification of PRCD mutations as the cause for retinal degeneration in dogs and humans [9,10]. The specific role played by PRCD in the retina was only recently discovered, as it was found by us and others to be essential for high-fidelity disc formation in both rod and cone photoreceptors [5,8]. Accordingly, PRCD is specifically located to photoreceptor OS [4,5]. PRCD anchoring to OS disc membranes is achieved by N-terminal post-translational modifications, including S-acylation and palmitoylation [6,7]. Moreover, a previous proteomic search for PRCD-interacting partners in disc membranes found that it binds rhodopsin. This interaction was critically important for supporting the intracellular stability of PRCD [7].

In the current study, we searched for additional PRCD-interacting proteins, and identified interactions with TULP1 and TUB. These two proteins, both members of the tubby-like family, are involved in diverse functions, including roles in energy balance (TUB), endocytosis (TULP1), synapse maintenance and architecture (TULP1), and stimulation of phagocytosis (TULP1 and TUB) (reviewed in reference [12]). However, one of their major roles is trafficking proteins into cilia. The primary cilium is an antenna-like cellular protrusion that mediates sensory and neuroendocrine signaling. The ciliary membrane is enriched with multiple integral membrane proteins. In the nervous system, TUB is involved in trafficking certain G protein-coupled receptors, including SSTR3, MCHR1, and NPY2R to neuronal cilia [20,28]. In the retina, both TULP1 and TUB are involved in the vesicular trafficking of unique OS proteins, including rhodopsin, GC1, GCAP1, GCAP2, cone opsins, and arrestin [15,18,19,20]. The current study adds PRCD to this list.

As indicated above, PRCD was shown to bind rhodopsin within the OS disc membrane [7]. As shown previously for rhodopsin, and as we show here for PRCD, trafficking of both proteins to the OS is dependent on TULP1 and TUB (Figure 5). Taken together, these findings raise the possibility that PRCD and rhodopsin may be co-transported to the OS and that their interaction occurs prior to their localization to the OS membrane.

TULP1 and TUB are two highly homologous proteins, both playing crucial roles in retinal function. Interestingly, these two proteins were found to interact with each other and to form heterodimers or heterooligomers. Their interaction was functionally revealed by their synergistic stimulation of phagocytosis of shed OS discs by retinal pigmented epithelium cells [29]. The fact that several proteins, including PRCD, depend on both TULP1 and TUB for their proper OS localization, and that the elimination of either one of them is sufficient to cause mislocalization of these proteins, indicates that TULP1 and TUB do not play a redundant role in OS protein localization. Moreover, it suggests that TULP1 and TUB cooperate in trafficking of photoreceptor proteins from the IS to the OS.

In summary, the findings described here enhance our understanding regarding the normal processing of PRCD within photoreceptor cells. They also add to our knowledge regarding the function of TULP1 and TUB and the retinal pathophysiology associated with deficiency of either one of these multi-functional proteins.

## 4. Materials and Methods

### 4.1. Ras-Recruitment System (RRS)

For the RRS screen, a bait plasmid expressing the murine PRCD protein as an N-terminal fusion to Myc-Ras was constructed in the plasmid pMet425 (Figure 1a) [11]. The bait expression was induced by the lack of methionine in the media (Figure 1b). The bait plasmid was co-transformed into the temperature sensitive yeast strain *cdc25-2* with a commercial bovine retinal cDNA library cloned into the CytoTrap pMyr XR vector (Stratagene, Agilent Technologies, Santa Clara, CA, USA). The presence of galactose in the media induced the expression of the library plasmid, in which clones were fused to Src myristoylation sequences. Approximately 350,000 colonies were screened. Transformed yeast were plated on solid synthetic media containing glucose and lacking leucine and uracil (GLU-LU), left to recover at 24 °C for 4–5 days, and then replicated to solid synthetic media containing galactose and lacking leucine, uracil, and methionine (GAL-LUM). Yeast colonies considered as putative positives were those which grew on GAL-LUM media at the restrictive temperature (36 °C). Colonies which were able to grow at 36 °C on GAL-LU plates (on which the bait protein was not expressed) were excluded from the screen. After this selection, library plasmids were isolated from positive clones and sequenced to reveal their identity. Specific interaction with PRCD was confirmed by re-transformation of positive plasmids into *cdc25-2* yeast with either PRCD or non-specific bait plasmids.

### 4.2. Cell Culture

COS-7 cells were grown in DMEM containing 10% fetal bovine serum, 1% penicillin/streptomycin, and 1% glutamine (Biological Industries, Beit Ha’Emek, Israel) at 37 °C and 5% CO_2_. Cells were transfected with different plasmid combinations using the jetPEI transfection reagent (Polyplus-transfection SA, Illkirch, France) and harvested at 48 h post transfection. COS-7 cells used in this study were authenticated as previously described [30].

### 4.3. Co-Immunoprecipitation (co-IP)

Mouse *Prcd* cDNA (NM_001163318) was cloned into the pCS2+MT expression vector [31], in frame with six C-terminal Myc tags. Full-length *Tulp1* (NM_021478.2) and *Tub* (NM_021885.4) cDNAs were cloned in frame with three N-terminal HA tags in the pcDNA-3HA expression vector (a gift from Prof. Ami Aronheim). Lysis of transfected cells was performed with WCE buffer (25 mM HEPES pH 7.7, 0.3 M NaCl, 1.5 mM MgCl_2_, 0.2 mM EDTA, 0.1% Triton X-100, 0.5 mM DTT, 20 mM β-glycerolphosphate, 0.1 mM Na_2_VO_4_, 100 µg/mL PMSF, protease inhibitor cocktail 1:100 (P8340, Sigma-Aldrich, St. Louis, MO, USA)). Protein extracts were incubated overnight at 4 °C with anti-Myc tag antibodies, followed by 1 h incubation with Protein A Sepharose beads (Sigma-Aldrich). Beads were washed four times with WCE buffer, and the precipitated proteins were eluted using SDS-PAGE sample buffer. For Western blot analysis, samples were subjected to denaturing polyacrylamide gel electrophoresis (SDS-PAGE) using 12.5% polyacrylamide gels, followed by transfer to nitrocellulose membranes (GE Healthcare, Buckinghamshire, UK). Membranes were incubated with a primary antibody, washed, and incubated with a peroxidase-conjugated secondary antibody. The Amersham ECL Western blotting analysis system (GE Healthcare) was used to visualize the results.

### 4.4. Pull-Down Assays

*Prcd* cDNA was cloned into the pGEX vector (GE Healthcare), in-frame with N-GST. PRCD-GST fusion protein was purified from *Escherichia coli* according to the manufacturer’s instructions. *Tulp1* and *Tub* cDNAs were cloned into the pcDNA-3HA expression vector and expressed in vitro using the TNT T7 Quick Coupled Transcription/Translation System (Promega, Madison, WI, USA). Various mutations were inserted using the quick change II site directed mutagenesis kit (Stratagene). For pull-down assays, in vitro translated TULP1 or TUB were mixed with 20 µg purified PRCD-GST beads and 300 µL pull-down buffer (20 mM Hepes, 100 mM NaCl, 1 mM DTT, 6 mM MgCl_2_, 20% glycerol, 1% NP-40, 0.5 mM EDTA). The solution was incubated for 1 h at room temperature and washed 4 times with pull-down buffer. Samples were boiled for 5 min and then processed for Western blot analysis with an anti-HA antibody.

### 4.5. Animal Experiments

The research was performed in adherence to the National Institute of Health’s *Guide for the Care and Use of Laboratory Animals* (NIH Publications No. 8023, revised 1978). The research was approved by the Animal Care and Use Committee at the Technion-Israel Institute of Technology (Protocol numbers IL-008-0119 and IL-114-0719). *Tulp1*-knockout mice (B6.129X1-Tulp1^tm1Pjn^/Pjn) [17] and *Tub*-mutant mice (CAST.B6-Tub^tub^/Jng) [32] were obtained from The Jackson Laboratory (USA).

### 4.6. Immunofluorescence

Mouse eyes were harvested in the light, fixed in 4% paraformaldehyde at 4 °C, cryoprotected in a graded sucrose series, and embedded in OCT buffer. Permeabilization of frozen sections (16 µm) was achieved with 1% triton X-100, followed by blocking in 5% fetal calf serum and overnight incubation at 4 °C with a primary antibody, followed by a secondary antibody. DAPI was used for nuclear staining. Examination of the stained sections was performed by an LSM 700 laser scanning confocal microscope (Carl Zeiss Meditec, Jena, Germany).

### 4.7. Antibodies

Primary antibodies used were mouse monoclonal antibody against HA tag (ab18181), rabbit polyclonal antibody against Myc tag (ab9106), mouse monoclonal antibody against rhodopsin (ab3267) (Abcam, Cambridge, MA, USA), and rabbit polyclonal antibody against PRCD [5]. Secondary antibodies used were peroxidase-conjugated AffiniPure goat anti-mouse IgG, peroxidase-conjugated AffiniPure goat anti-rabbit IgG, Cy3-conjugated donkey anti-mouse IgG, Cy3-conjugated donkey anti-rabbit IgG (Jackson ImmunoResearch Laboratories, West Grove, PA, USA), and GST HRP rabbit polyclonal IgG (Santa Cruz Biotechnology, Dallas, TX, USA).

## Abbreviations

Co-IP	co-immunoprecipitation
GC1	guanylate cyclase 1
GCAP1	guanylate cyclase activating protein
IS	inner segment
ONL	outer nuclear layer
OPL	outer plexiform layer
OS	outer segment
PRCD	Photoreceptor Disc Component
RP	Retinitis pigmentosa
RRS	Ras-Recruitment System
TULP1	Tubby-like protein 1

## References

[1] May-Simera H., Nagel-Wolfrum K., Wolfrum U. (2017). Cilia—The sensory antennae in the eye. Prog. Retin. Eye Res..

[2] Kevany B.M., Palczewski K. (2010). Phagocytosis of retinal rod and cone photoreceptors. Physiology.

[3] Salinas R.Y., Pearring J.N., Ding J.D., Spencer W.J., Hao Y., Arshavsky V.Y. (2017). Photoreceptor discs form through peripherin-dependent suppression of ciliary ectosome release. J. Cell Biol..

[4] Skiba N.A., Spencer W.J., Salinas R.Y., Lieu E.C., Thompson J.W., Arshavsky V.Y. (2013). Proteomic identification of unique photoreceptor disc components reveals the presence of PRCD, a protein linked to retinal degeneration. J. Proteome Res..

[5] Allon G., Mann I., Remez L., Sehn E., Rizel L., Nevet M.J., Perlman I., Wolfrum U., Ben-Yosef T. (2019). PRCD is concentrated at the base of photoreceptor outer segments and is involved in outer segment disc formation. Hum. Mol. Genet..

[6] Murphy J., Kolandaivelu S. (2016). Palmitoylation of Progressive Rod-Cone Degeneration (PRCD) Regulates Protein Stability and Localization. J. Biol. Chem..

[7] Spencer W.J., Pearring J.N., Salinas R.Y., Loiselle D.R., Skiba N.P., Arshavsky V.Y. (2016). Progressive Rod-Cone Degeneration (PRCD) Protein Requires N-Terminal S-Acylation and Rhodopsin Binding for Photoreceptor Outer Segment Localization and Maintaining Intracellular Stability. Biochemistry.

[8] Spencer W.J., Ding J.D., Lewis T.R., Yu C., Phan S., Pearring J.N., Kim K.Y., Thor A., Mathew R., Kalnitsky J. (2019). PRCD is essential for high-fidelity photoreceptor disc formation. Proc. Natl. Acad. Sci. USA.

[9] Zangerl B., Goldstein O., Philp A.R., Lindauer S.J., Pearce-Kelling S.E., Mullins R.F., Graphodatsky A.S., Ripoll D., Felix J.S., Stone E.M. (2006). Identical mutation in a novel retinal gene causes progressive rod-cone degeneration in dogs and retinitis pigmentosa in humans. Genomics.

[10] Nevet M.J., Shalev S.A., Zlotogora J., Mazzawi N., Ben-Yosef T. (2010). The identification of a prevalent founder mutation in an Israeli Muslim Arab village confirms the role of *PRCD* in the etiology of retinitis pigmentosa in humans. J. Med. Genet..

[11] Aronheim A. (2018). The Ras Recruitment System (RRS) for the Identification and Characterization of Protein-Protein Interactions. Methods Mol. Biol..

[12] Wang M., Xu Z., Kong Y. (2018). The tubby-like proteins kingdom in animals and plants. Gene.

[13] Banerjee P., Kleyn P.W., Knowles J.A., Lewis C.A., Ross B.M., Parano E., Kovats S.G., Lee J.J., Penchaszadeh G.K., Ott J. (1998). *TULP1* mutation in two extended Dominican kindreds with autosomal recessive retinitis pigmentosa. Nat. Genet..

[14] Borman A.D., Pearce L.R., Mackay D.S., Nagel-Wolfrum K., Davidson A.E., Henderson R., Garg S., Waseem N.H., Webster A.R., Plagnol V. (2014). A homozygous mutation in the *TUB* gene associated with retinal dystrophy and obesity. Hum. Mutat..

[15] Hagstrom S.A., Duyao M., North M.A., Li T. (1999). Retinal degeneration in *tulp1*-/- mice: Vesicular accumulation in the interphotoreceptor matrix. Investig. Ophthalmol. Vis. Sci..

[16] Hagstrom S.A., North M.A., Nishina P.L., Berson E.L., Dryja T.P. (1998). Recessive mutations in the gene encoding the tubby-like protein TULP1 in patients with retinitis pigmentosa. Nat. Genet..

[17] Ikeda S., Shiva N., Ikeda A., Smith R.S., Nusinowitz S., Yan G., Lin T.R., Chu S., Heckenlively J.R., North M.A. (2000). Retinal degeneration but not obesity is observed in null mutants of the tubby-like protein 1 gene. Hum. Mol. Genet..

[18] Grossman G.H., Watson R.F., Pauer G.J., Bollinger K., Hagstrom S.A. (2011). Immunocytochemical evidence of Tulp1-dependent outer segment protein transport pathways in photoreceptor cells. Exp. Eye Res..

[19] Hagstrom S.A., Adamian M., Scimeca M., Pawlyk B.S., Yue G., Li T. (2001). A role for the Tubby-like protein 1 in rhodopsin transport. Investig. Ophthalmol. Vis. Sci..

[20] Sun X., Haley J., Bulgakov O.V., Cai X., McGinnis J., Li T. (2012). Tubby is required for trafficking G protein-coupled receptors to neuronal cilia. Cilia.

[21] Aronheim A., Broder Y.C., Cohen A., Fritsch A., Belisle B., Abo A. (1998). Chp, a homologue of the GTPase Cdc42Hs, activates the JNK pathway and is implicated in reorganizing the actin cytoskeleton. Curr. Biol..

[22] Remez L., Zobor D., Kohl S., Ben-Yosef T. (2014). The progressive rod-cone degeneration (PRCD) protein is secreted through the conventional ER/Golgi-dependent pathway. Exp. Eye Res..

[23] Gu S., Lennon A., Li Y., Lorenz B., Fossarello M., North M., Gal A., Wright A. (1998). Tubby-like protein-1 mutations in autosomal recessive retinitis pigmentosa. Lancet.

[24] Hanein S., Perrault I., Gerber S., Tanguy G., Barbet F., Ducroq D., Calvas P., Dollfus H., Hamel C., Lopponen T. (2004). Leber congenital amaurosis: Comprehensive survey of the genetic heterogeneity, refinement of the clinical definition, and genotype-phenotype correlations as a strategy for molecular diagnosis. Hum. Mutat..

[25] Hebrard M., Manes G., Bocquet B., Meunier I., Coustes-Chazalette D., Herald E., Senechal A., Bolland-Auge A., Zelenika D., Hamel C.P. (2011). Combining gene mapping and phenotype assessment for fast mutation finding in non-consanguineous autosomal recessive retinitis pigmentosa families. Eur. J. Hum. Genet..

[26] Kannabiran C., Singh H., Sahini N., Jalali S., Mohan G. (2012). Mutations in *TULP1, NR2E3*, and *MFRP* genes in Indian families with autosomal recessive retinitis pigmentosa. Mol. Vis..

[27] Kondo H., Qin M., Mizota A., Kondo M., Hayashi H., Hayashi K., Oshima K., Tahira T., Hayashi K. (2004). A homozygosity-based search for mutations in patients with autosomal recessive retinitis pigmentosa, using microsatellite markers. Investig. Ophthalmol. Vis. Sci..

[28] Loktev A.V., Jackson P.K. (2013). Neuropeptide Y family receptors traffic via the Bardet-Biedl syndrome pathway to signal in neuronal primary cilia. Cell Rep..

[29] Caberoy N.B. (2014). Synergistic interaction of tubby and tubby-like protein 1 (Tulp1). Adv. Exp. Med. Biol..

[30] Almeida J.L., Hill C.R., Cole K.D. (2011). Authentication of African green monkey cell lines using human short tandem repeat markers. BMC Biotechnol..

[31] Roth M.B., Zahler A.M., Stolk J.A. (1991). A conserved family of nuclear phosphoproteins localized to sites of polymerase II transcription. J. Cell Biol..

[32] Coleman D.L., Eicher E.M. (1990). Fat (fat) and tubby (tub): Two autosomal recessive mutations causing obesity syndromes in the mouse. J. Hered..

